# The Effect of Exercise Atmosphere on College Students’ Physical Exercise—A Moderated Chain Mediation Model

**DOI:** 10.3390/bs15040507

**Published:** 2025-04-10

**Authors:** Ting Zhang, Bowen Li, Xinqi He, Peng Jia, Zicong Ye

**Affiliations:** 1 Physical Education College, Jiangxi Normal University, Nanchang 330022, China; 2 Department of Physical Education, Dongbei University of Finance and Economics, Dalian 116025, China; 3 School of Physical Education and Sport, Wuhan University of Science and Technology, Wuhan 430081, China

**Keywords:** exercise atmosphere, physical exercise, social physique anxiety, enjoyment, exercise

## Abstract

To explore the influence of exercise atmosphere on college students’ physical exercise and its mechanism of action, the Exercise Atmosphere Scale, Enjoyment Scale, Exercise Self-efficacy Scale, Social Physique Anxiety Scale, and Physical Exercise Rating Scale were used to investigate 1265 college students. The results showed that: (1) exercise atmosphere directly impacted physical exercise and indirectly influenced physical exercise through three mediating pathways (e.g., the mediating role of enjoyment, the mediating role of exercise self-efficacy, and the chain mediating role of enjoyment and exercise self-efficacy); (2) social physique anxiety negatively moderated the influence of exercise atmosphere on enjoyment and exercise self-efficacy, respectively, and negatively moderated three mediating pathways of exercise atmosphere influencing physical exercise through enjoyment and exercise self-efficacy. The results are helpful in enriching the research on exercise atmosphere and college students’ physical exercise and provide useful insights for schools and related organizations to strengthen college students’ physical exercise.

## 1. Introduction

Physical exercise represents the expansion and extension of physical education, encompassing a variety of physical activities that students engage in through various means. The aim is to promote health, entertainment, and recreation, primarily utilizing extracurricular leisure time. This is mainly reflected in aspects such as frequency, intensity, and duration (Cheng & Dong, 2018). In recent years, the lack of participation in physical exercise among college students has become a growing concern for all sectors of society (B. L. Dong & Mao, 2018). Although colleges generally offer physical education courses and are equipped with sports facilities, many students still lack regular physical exercise habits, and sedentary behavior is more common (Garn & Simonton, 2022). Studies have shown that individual exercise behavior is not only influenced by internal factors such as personal motivation and attitude (Su et al., 2024) but is also closely related to the external environment, in which exercise atmosphere (e.g., peer support, club activities, etc.) is considered a key social environmental factor (Cheng & Dong, 2018). A good exercise atmosphere may indirectly promote college students’ physical exercise through motivation and self-efficacy (W. N. Liu et al., 2011). However, the specific mechanism of this effect is unclear, and the heterogeneous effects of boundary conditions have been less examined. Therefore, this study intends to construct a moderated chain-mediated model to investigate how exercise atmosphere affects college students’ physical exercise through multiple mediating pathways and to analyze the moderating role of individual factors.

### 1.1. The Relationship Between Exercise Atmosphere and Physical Exercise

In recent years, scholars generally agree that “exercise atmosphere is an external resource for the generation, maintenance, and development of autonomous behaviors of physical exercise” (S. P. Chen & Li, 2007). Exercise atmosphere is the physical exercise environment created by the surrounding people and the information about physical exercise available to the participants, which mainly includes the natural atmosphere and interpersonal atmosphere, and it has a certain motivating and guiding effect on physical exercise (Y. Q. Dong et al., 2023). A positive exercise atmosphere is conducive to promoting college students’ emotional communication and improving their social skills, considering physical exercise as a social activity to enjoy their body and mind and realize their self-worth, and better motivating college students to participate in physical exercise (Su et al., 2024). Exercise atmosphere has a certain influence on college students’ awareness and concept of exercise, which is an exogenous motivation to promote adherence to physical exercise. It can also play the role of peer example to motivate and guide individuals to form positive exercise behaviors (Su et al., 2024). A poor exercise atmosphere can constrain college students’ desire to exercise and even cause individuals to interrupt or withdraw from physical exercise (B. L. Dong & Mao, 2018). Cheng and Dong (2018) found that the exercise atmosphere can directly influence college students to engage in leisure physical exercise. B. L. Dong and Mao (2018) found that exercise atmosphere had a direct positive impact on college students’ exercise persistence. A good exercise atmosphere will provide a decision-making basis when individuals decide to exercise or not, mobilize ruminative thinking based on the exercise behaviors and performances of peers around them, and engage in physical exercise under the demonstration and leadership of peers (B. L. Dong & Mao, 2018). In conclusion, both the natural atmosphere and the interpersonal atmosphere should be important external factors to motivate college students to engage in physical exercise independently and actively in their spare time.

### 1.2. The Mediating Role of Enjoyment

Enjoyment is understood as the subjective experience of pleasure and satisfaction during physical exercise (Nielsen et al., 2014). The natural conditions of the exercise atmosphere, such as the venue and equipment, predict the opportunities, conditions, and satisfaction of physical exercise, which benefits the enrichment of the exerciser’s experience of pleasure, feelings, positive emotions, and desire to participate, and becomes an informational memory for decision-making about physical exercise; this makes it possible for the individual to maintain regular, frequent, and autonomous physical exercise (Bower, 1981). In addition, enjoyment can greatly influence an individual’s perception of physical exercise, thereby enhancing it (when perceived as fun or enjoyable) or avoiding it (when perceived as unpleasant, uninteresting, or boring), influencing physical exercise participation (Teixeira et al., 2021, 2022). Recent related theories emphasize enjoyment as a relevant factor that can explain and support physical activity. For example, based on the dual-process theory, Brand and Ekkekakis (2018) proposed affective-reflective theory (ART), which emphasizes that when people experience pleasure, they tend to repeat the behavior (exercise), but that unpleasant affective experiences also reduce the probability of repetition. As explained by the emotional memory theory, emotions triggered by the social environment (pleasantness, uneasiness, fear, anger, etc.) become informational symbols of the subject’s cognitive memory, which reconstructs the cognitive system and guides future behaviors (MeAuley & Courneya, 1994). In addition, the interpersonal supportive exercise atmosphere conveys emotional care for the subject, which can motivate individuals to display themselves courageously and establish group identity, so that they can fully release their emotions and stimulate enjoyment experiences in practice, which in turn promotes physical exercise (Cheng & Dong, 2018). Therefore, college students’ exercise atmosphere may indirectly affect physical exercise through enjoyment.

### 1.3. The Mediating Role of Exercise Self-Efficacy

Self-efficacy is a central concept in social cognitive theory (SCT), which focuses on an individual’s perception of the relationship between situational demands and personal capabilities, and is often used as an important variable in exploring engagement in behavior change (Bandura, 1997). Several studies have shown that self-efficacy is significantly correlated with behavior (Bandura, 1997). Exercise self-efficacy refers to an individual’s confidence or belief in his or her ability to perform physical exercise consistently and regularly in order to achieve a certain behavioral outcome (maintaining physical and mental health, improving body shape, etc.) (Yan et al., 2019). The exercise atmosphere has a better predictive effect on college students’ exercise self-efficacy. In a good interpersonal exercise atmosphere, when students face challenges or are emotionally exhausted during physical exercise, they can get help and support from teachers and parents, and the mutual emotional communication among peers is conducive to completing the exercise task in a timely manner and maintaining emotional stability, which in turn improves students’ exercise self-efficacy (Y. Li et al., 2024). In addition, exercise self-efficacy has an important impact on exercise behavior, which helps optimize cognitive processes and can determine the selectivity and persistence of exercise behavior (Yang, 2013). Studies have shown that people with high exercise efficacy sense have more persistent leisure exercise, more reasonable exercise intensity, more regular exercise behavior, and more autonomy of behavior (Y. Q. Dong et al., 2022). In addition, scholars have also demonstrated that self-efficacy plays a mediating role in the influence of exercise atmosphere on physical exercise (Y. Q. Dong et al., 2022; Y. Li et al., 2024). Therefore, college students’ exercise atmosphere may indirectly influence physical exercise through exercise self-efficacy.

### 1.4. The Chain Mediating Role of Enjoyment and Exercise Self-Efficacy

Not only can enjoyment and exercise self-efficacy influence physical exercise, but they also interact with each other. Increased enjoyment should directly lead to increased physical activity (Vallerand et al., 1987). Enjoyment may also have an indirect effect on physical activity by influencing self-efficacy. According to social cognitive theory, self-efficacy is the closest influence on behavior and has been suggested to mediate the effects of affective states on behavior (Bandura, 1997). Affective responses related to the enjoyment of physical activity are a source of information about self-efficacy (Bandura, 1997) and influence the mediating effect of self-efficacy on changes in physical activity (Dishman et al., 2005). Previous studies have reported significant indirect effects of emotional responses or exercise enjoyment on physical activity by influencing self-efficacy in adolescents and older adults (H. Chen et al., 2017; McAuley et al., 2003). It is inferred that enjoyment may also have an indirect effect on physical exercise through exercise self-efficacy. In addition, a positive exercise atmosphere promotes individuals to form effective interactions with the surrounding environment and others, which is conducive to obtaining a positive enjoyment experience, promotes the enhancement of individual exercise self-efficacy, regards physical exercise as a social activity that pleases the body and mind, realizes self-worth, and better motivates college students to participate in physical exercise (Y. Q. Dong et al., 2022). Therefore, college students’ exercise atmosphere may indirectly affect physical exercise through the chain mediation of enjoyment and exercise self-efficacy.

### 1.5. The Moderating Role of Social Physique Anxiety

Social physique anxiety is the level of anxiety experienced by individuals when faced with others’ evaluations of their physique (Hart et al., 1989). It stems from an individual’s uncertainty about his or her self-performance of physique and concern about evaluation by others (Hart et al., 1989). Self-performance theory emphasizes the individual’s efforts to control and manage the impression he or she makes on others in social settings (Leary, 1992). In physical exercise situations, individuals may worry that their physical performance does not meet social expectations or the evaluative standards of others, resulting in anxiety (Crawford & Eklund, 1994). This anxiety may affect an individual’s participation in physical exercise, preventing the individual from interacting effectively with the surrounding environment and others, and leading to a decrease in the overall activity level (Crawford & Eklund, 1994). At the same time, individuals may be overly concerned with self-expression, which may reduce intrinsic motivation for physical exercise, and rely on external motivation (e.g., weight loss, shaping, etc.), which may not be conducive to the development of long-term exercise habits (Brown, 2000), further affecting the sustainability of the exercise atmosphere. In addition, during exercise, individuals high in social physique anxiety may be constantly preoccupied with their size and performance, worrying about the perceptions and evaluations of others (Frederick & Morrison, 1996). This distraction not only affects the exercise atmosphere, but also makes it difficult for individuals to focus on the fun and enjoyment of exercise. They may not be able to fully experience the physical and mental relaxation and pleasure of exercise because anxiety is always predominant (Frederick & Morrison, 1996). Related research has found that even after completing exercise, individuals high in social physique anxiety may be more reluctant to participate in exercise due to dissatisfaction with their performance that diminishes their positive enjoyment of the exercise experience (Sabiston et al., 2014), coupled with the activation of the psychological defense mechanism of physique anxiety, i.e., avoidance of exercise to avoid compromising their social self-esteem in the exercise setting (Brown, 2000). Thus, social physique anxiety may modulate the effect of exercise atmosphere on enjoyment and the mediating effect of enjoyment between exercise atmosphere and college students’ physical exercise.

Individuals with high social physique anxiety are often accompanied by feelings of dissatisfaction with their bodies, and this dissatisfaction reduces an individual’s self-efficacy for exercise, as they may believe that they are unable to achieve their ideal physique through exercise (Rothberger, 2014). Social physique anxiety can also trigger social comparisons in which individuals are at a disadvantage compared to others, leading to a devaluation of an individual’s exercise self-efficacy (Fitzsimmons-Craft et al., 2012). Related research has also found that levels of exercise self-efficacy decrease with increasing social physique anxiety in both males and females, regardless of the exercise setting (Rothberger et al., 2015). Self-efficacy theory suggests that individuals tend to engage in activities that make them feel empowered and increase their self-confidence, including personal health habits (Bandura, 1997). As anxiety increases, individuals may attempt to avoid uncertainty about skill competence or coping ability, reducing physical exercise (Sabiston et al., 2014). Thus, social physique anxiety may modulate the effects of exercise atmosphere on exercise self-efficacy and the mediating effect of exercise self-efficacy between exercise atmosphere and college students’ physical exercise.

Under sociocultural pressures of appearance (Ajibewa et al., 2024), social physique anxiety due to the fear of negative appearance evaluations in exercise settings causes individuals to choose to reduce their level of physical exercise participation to escape from the threatening and hurtful messages conveyed by external physical appearance evaluations and reduces the individual’s opportunities for contact with the natural and interpersonal environments (Nutter et al., 2021), thereby reducing the overall exercise atmosphere. At the same time, the inability to obtain the help and feedback provided by the interpersonal atmosphere will cause individuals to amplify negative exercise emotions such as fear, uneasiness, and dread, masking or diminishing the enjoyment experience of exercise (Guo et al., 2023), which in turn will lead to self-doubt and self-depreciation of one’s own exercise ability (Pearl et al., 2021), which will result in further coping responses that reduce physical exercise ways (Anić et al., 2022). Thus, social physique anxiety may modulate the chain-mediated effects of enjoyment and exercise self-efficacy between exercise atmosphere and college students’ physical exercise.

### 1.6. The Present Study

In summary, although many studies have examined the influence of exercise atmosphere on college students’ physical exercise, few studies have explored in depth the mechanism of exercise atmosphere’s influence on students’ physical exercise. In addition, although previous studies have found that both enjoyment and exercise self-efficacy are effective mediators that positively influence college students’ physical exercise, few studies have incorporated these two mediators sequentially into the model of the influence of exercise atmosphere on college students’ physical exercise to conduct multiple mediation analysis. Finally, most of the studies have used social physique anxiety as an independent or mediating variable rather than as a moderating variable.

**Figure 1:**
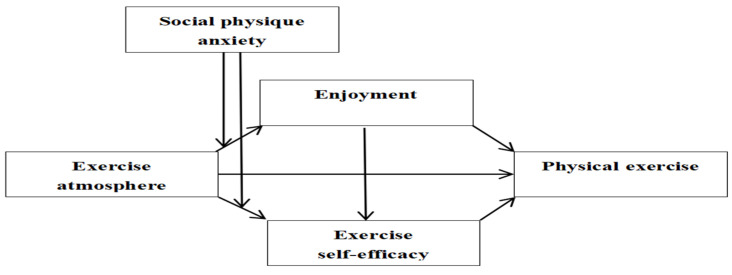
Moderated mediation model of the effect of exercise atmosphere on physical exercise.

Mainly based on social cognitive theory, combined with affective-reflective theory, self-performance theory, and previous research findings, this study proposes a moderated chain mediation model (see Figure 1) to present the following research hypotheses: Hypothesis 1. Exercise atmosphere has a positive and direct effect on college students’ physical exercise; Hypothesis 2. Enjoyment mediates the effect of exercise atmosphere on college students’ physical exercise; Hypothesis 3. Exercise self-efficacy mediates the effect of exercise atmosphere on college students’ physical exercise; Hypothesis 4. Enjoyment (first mediator) and exercise self-efficacy (second mediator) sequentially mediate the effect of exercise atmosphere on college students’ physical exercise. Hypothesis 5. Social physique anxiety negatively moderates the positive effect of exercise atmosphere on enjoyment; Hypothesis 6. Social physique anxiety negatively moderates the positive effect of exercise atmosphere on exercise self-efficacy; Hypothesis 7. Social physique anxiety negatively moderates the mediating effect of enjoyment between exercise atmosphere and college students’ physical exercise; Hypothesis 8. Social physique anxiety negatively moderates the mediating effect of exercise self-efficacy between exercise atmosphere and college students’ physical exercise; Hypothesis 9. Social physique anxiety negatively moderates the chain mediating effect of enjoyment and exercise self-efficacy between exercise atmosphere and college students’ physical exercise.

## 2. Materials and Methods

### 2.1. Participants

Using the whole cluster sampling method, some freshman and sophomore undergraduate students in seven universities in Jiangxi Province were selected in May and June 2024 by class for the questionnaire survey and aerobic fitness test. Prior to the formal survey and test, the researcher explained to the students about the subjects’ informed consent for this study, and after obtaining the students’ informed consent, the related survey and test were started. The sample size of the questionnaire data was 1864. The invalid questionnaires were eliminated due to highly repeated answers, reverse question quizzes, and the time taken to fill in the questionnaires, etc., and there were 1265 valid samples. Among them, 506 (40.0%) were male and 759 (60.0%) were female. Among them, 642 (50.8%) were rural and 623 (49.2%) were urban. There were 778 (61.5%) freshmen and 487 (38.5%) sophomores. The last physical fitness test scores were as follows: 222 college students (17.6%) with a score of 60 or less, 396 college students (31.3%) with a score of 61–70, 386 college students (30.5%) with a score of 71–80, 172 college students (13.6%) with a score of 81–90, and 89 college students (7.0%) with a score of 91–100. College students and their parents provided written informed consent for this study.

### 2.2. Measures

#### 2.2.1. Social Physique Anxiety Scale

The social physique anxiety scale (SPAS), revised by Xu (2003), was used. A total of 14 items were included, which were mainly related to three dimensions: concern about negative evaluations by others, discomfort with self-presentation of physique, and uneasiness with social comparisons. For example, “I feel relaxed and comfortable even if others do not rate my size or physical appearance highly.” All items were measured on a five-point Likert scale ranging from “1” (not at all) to “5” (completely). Scores on each item of the scale were summed, with higher total scores indicating higher levels of social and physical anxiety among college students. A confirmatory factor analysis (CFA) yielded acceptable fit indicators (NFI = 0.896, GFI = 0.923, CFI = 0.905, RMSEA = 0.084) of the scale in this study. The Cronbach’s alpha coefficient for the total scale was 0.877. 

#### 2.2.2. Enjoyment Scale

Enjoyment scale using the achievement emotions questionnaire (AEQ; Pekrun et al., 2005). The term “physical activity” in the scale was revised to “physical exercise,” e.g., “I feel pleasure in physical exercise.” There are four items with one dimension, and all items are measured on a five-point Likert scale ranging from “1” (not at all) to “5” (completely). The scale is reliable for college students (Garn & Simonton, 2022). The scores for each item of the scale were summed, with higher total scores indicating higher enjoyment by college students. A confirmatory factor analysis (CFA) yielded acceptable fit indicators (NFI = 0.998, GFI = 0.997, CFI = 0.998, RMSEA = 0.043) of the scale in this study. The cronbach’s alpha coefficient for the scale was 0.885.

#### 2.2.3. Exercise Self-Efficacy Scale

The exercise self-efficacy scale compiled by Wu et al. (2002) was used and combined with the research of S. Wang et al. (2016) to amend and compile, e.g., “I will insist on exercising even if I feel tired.” There are 12 items with one dimension, and all items are measured on a three-point Likert scale from “1” (I can’t do it) to “3” (I am sure I can do it), and the scores of each item of the scale are summed up, and the higher the total score, the higher the college students’ self-efficacy in exercise. A confirmatory factor analysis (CFA) yielded acceptable fit indicators (NFI = 0.933, GFI = 0.938, CFI = 0.940, RMSEA = 0.077) of the scale in this study. The Cronbach’s alpha coefficient of the scale was 0.907.

#### 2.2.4. Exercise Atmosphere Scale

Adopting the outdoor exercise atmosphere scale revised by W. N. Liu et al. (2011), the term “outdoor exercise” in the scale was revised to “physical exercise,” e.g., “In physical exercise, I have received help from my partners.” A total of 11 items were included, which were mainly related to three dimensions: interpersonal associations, natural associations, and information access. All items were measured using a five-point Likert scale ranging from “1” (completely disagree) to “5” (completely agree), and the scores of each item on the scale were summed, with higher total scores indicating a higher atmosphere for exercise among college students. A confirmatory factor analysis (CFA) yielded acceptable fit indicators (NFI = 0.949, GFI = 0.966, CFI = 0.958, RMSEA = 0.062) of the scale in this study. The Cronbach’s alpha coefficient of the scale was 0.844. 

#### 2.2.5. Physical Exercise Rating Scale

The physical exercise rating scale developed by Liang (1994) was used to examine the physical exercise of college students in terms of three items: intensity, frequency, and time of exercise. Physical exercise = intensity × (time − 1) × frequency. Intensity, time, and frequency were divided into 5 grades, which were scored from 1 to 5, with a maximum score of 100 and a minimum score of 0, respectively. The retest reliability of the scale was 0.82 (Liang, 1994). 

### 2.3. Data Analysis

SPSS 20.0 was used to conduct reliability tests, common method bias tests, descriptive statistical analyses, and correlation analyses of the scales. Model 6 and Model 84 in the Process macro program version 3.5, written by Hayes (2013), were calculated for chain mediation and moderated chain mediation (*p* < 0.05), respectively, using the bias-corrected nonparametric percentile Bootstrap method [5000 repetitions, 95% CIs]. The mediating effects were statistically significant if the path factor did not contain 0 within the confidence interval. 

Demographic variables were converted to include height, weight, parents’ educational qualifications, and monthly household income. In the questionnaire, height and weight were converted to Body Mass Index (BMI) according to weight (kg)/height^2^ (m). Referring to the calculation method of Fang et al. (2008), the variables of parents’ education level and monthly family income were assigned different weights, and then the principal component method was used to form the family socioeconomic status (SES) index.

## 3. Results

### 3.1. Common Method Deviation Test

The data used in the study were obtained from paper-based questionnaires, which may be subject to common method bias. Harman’s one-factor method was used to test for common method bias, and a non-rotated principal component factor analysis was performed on all question items. The results showed that eight factors had initial eigenvalues greater than 1, and the variance explained by the first factor was 21.47%, which was lower than the critical value of 40%. This indicates that the common method bias of this study is not significant. 

### 3.2. Descriptive Statistics and Correlation Analysis

As shown in Table 1, social physique anxiety was significantly negatively correlated with enjoyment, exercise self-efficacy, physical exercise, and exercise atmosphere; enjoyment was significantly positively correlated with exercise self-efficacy, physical exercise, and exercise atmosphere; exercise self-efficacy was significantly positively correlated with physical exercise and exercise atmosphere; and exercise atmosphere and physical exercise were significantly positively correlated. In addition, social physique anxiety was significantly positively correlated with gender and BMI, and significantly negatively correlated with SES; enjoyment was significantly negatively correlated with gender and significantly positively correlated with the physical fitness test; exercise self-efficacy was significantly negatively correlated with gender and significantly positively correlated with the physical fitness test and SES; exercise atmosphere was significantly positively correlated with the physical fitness test; and physical exercise was significantly negatively correlated with gender and significantly positively correlated with the physical fitness test and SES.

### 3.3. Moderated Mediation Model Testing

All relevant variables were standardized before conducting the correlation test according to the suggestion of Wen and Ye (2014). The moderated chain mediation effect was tested in two steps after controlling for the coordinating variables, such as gender, physical fitness test, SES, and BMI.

In the first step, Model 6 of the macro program PROCESS in SPSS 20.0 was used to test the mediating effect of enjoyment and exercise self-efficacy between exercise atmosphere and physical exercise, controlling for the relevant covariates. The results of the regression analysis showed (see Table 2) that exercise atmosphere significantly and positively predicted enjoyment (β = 0.494, *p* < 0.001), exercise self-efficacy (β = 0.155, *p* < 0.001), and physical exercise (β = 0.064, *p* < 0.05), respectively; and enjoyment significantly and positively predicted exercise self-efficacy (β = 0.441, *p* < 0.001) and physical exercise (β = 0.155, *p* < 0.001), respectively; and exercise self-efficacy significantly and positively predicted physical exercise (β = 0.146, *p* < 0.001). 

First, as can be seen from the mediation effects analysis (see Table 3), the total effect of exercise atmosphere on physical exercise was significant (effect size = 0.195; Boot95% CI [0.139, 0.249]). Second, the direct effect of the exercise atmosphere on physical exercise was significant (effect size = 0.064; Boot95% CI [0.008, 0.123]), indicating that Hypothesis 1 was supported. Third, results showed a significant mediating effect of exercise atmosphere on physical exercise through either enjoyment (effect size = 0.076; Boot95% CI [0.039, 0.115]) or exercise self-efficacy (effect size = 0.023; Boot95% CI [0.010, 0.039]), supporting both Hypotheses 2 and 3. Finally, the mediating effect of exercise atmosphere on physical exercise through the chain mediation of enjoyment and exercise self-efficacy was significant (effect size = 0.032; Boot95% CI [0.018, 0.048]), which supported Hypothesis 4. 

**Figure 2:**
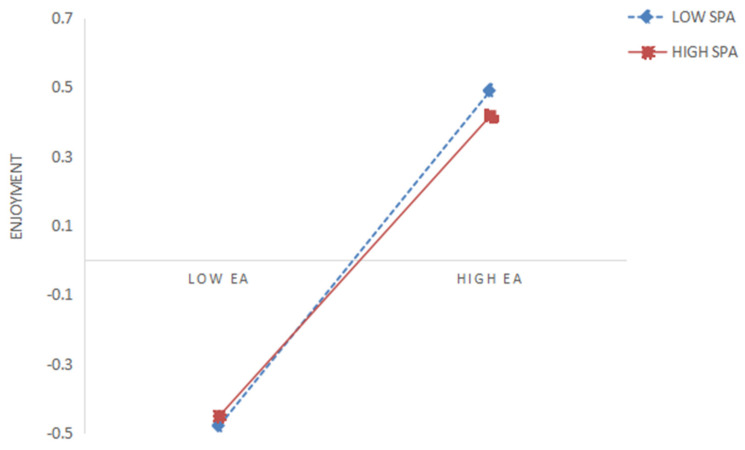
Interaction effect of EA with SPA on enjoyment.

**Figure 3:**
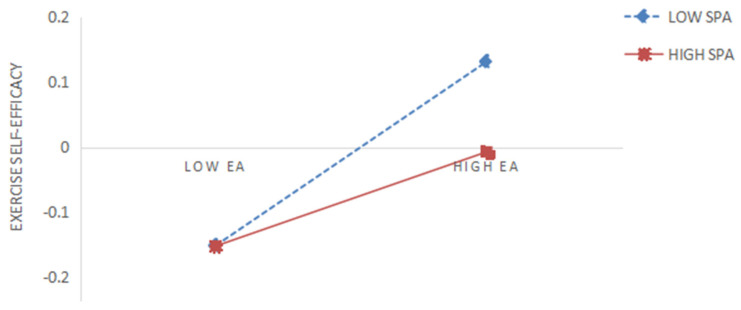
Interaction effect of EA with SPA on exercise self-efficacy.

In the second step, Model 84 of the macro program PROCESS in SPSS 20.0 was used to test the moderating effect of social physique anxiety, controlling for relevant covariates. The results showed (see Table 4) that the product term of exercise atmosphere and social physique anxiety was a significant negative predictor of both enjoyment and exercise self-efficacy (β = −0.051, *p* < 0.05; β = −0.069, *p* < 0.01), which was opposite the sign of the regression coefficients of exercise atmosphere on enjoyment and exercise self-efficacy, respectively, suggesting that the higher the social physique anxiety, the weaker the predictive effect of exercise atmosphere on enjoyment and exercise self-efficacy, respectively. Therefore, both Hypotheses 5 and 6 proposed in this study were validated. In order to further reveal the moderating role of social physique anxiety in the relationship between exercise atmosphere and enjoyment and exercise self-efficacy, the present study grouped social physique anxiety into high and low groups according to the mean plus or minus one standard deviation and performed a simple slope test. As can be seen in Figure 2, for college students with lower social physique anxiety (M − 1SD), exercise atmosphere significantly predicted enjoyment, b_simple_ = 0.535, *p* < 0.001. However, for college students with higher social physique anxiety (M + 1SD), exercise atmosphere significantly predicted enjoyment but much weaker, b_simple_ = 0.433, *p* < 0.001, indicating a weakening effect of social physique anxiety. As shown in Figure 3, for college students with lower social physique anxiety (M − 1SD), exercise atmosphere significantly predicted exercise self-efficacy, b_simple_ = 0.211, *p* < 0.001. However, for college students with higher social physique anxiety (M + 1SD), exercise atmosphere did not significantly predict exercise self-efficacy but was much weaker, b_simple_ = 0.072, *p* < 0.05, indicating a weakening effect of social physique anxiety.

A difference-in-differences grouping method (Edwards & Lambert, 2007) was used to test for a moderated mediating effect, and the results are shown in Table 5. The mediating effect of exercise atmosphere on physical exercise through enjoyment was significant (effect size = 0.067; Boot95% CI [0.034, 0.104]) when social physique anxiety was high (M + 1SD) and was significant (effect size = 0.083; Boot95% CI [0.042, 0.127]) when social physique anxiety was low (M − 1SD). The between-group difference was significant (effect size = −0.016; Boot95% CI [−0.035, −0.001]). Thus, H7 was supported. Similarly, the mediating effect of exercise atmosphere on physical exercise through exercise self-efficacy was not significant (effect size = 0.011; Boot95% CI [−0.002, 0.025]) when social physique anxiety was high (M + 1SD) and was significant (effect size = 0.031; Boot95% CI [0.014, 0.052]) when social physique anxiety was low (M − 1SD). The between-group difference was significant (effect size = −0.020; Boot95% CI [−0.041, −0.005]). H8 was supported. In addition, the chain-mediated effect of exercise atmosphere on physical exercise through enjoyment and exercise self-efficacy was significant (effect size = 0.027; Boot95% CI [0.014, 0.043]) when social physique anxiety was high (M + 1SD) and was significant (effect size = 0.034; Boot95% CI [0.018, 0.052]) when social physique anxiety was low (M − 1SD). The between-group difference was significant (effect size = −0.007; Boot95% CI [−0.015, −0.001]). Thus, H9 was also supported.

## 4. Discussion

### 4.1. Influence of Exercise Atmosphere on Physical Exercise

Correlation and regression analyses showed that exercise atmosphere helps to stimulate college students’ physical exercise, and this result is consistent with previous opinions (Cheng & Dong, 2018). Research reports that reasonable site planning, adequate venue resources, and an appropriate environmental atmosphere help to improve the level of accessibility and accessibility of students’ exercise, stimulate students’ desire to exercise (Cheng & Dong, 2018). At the same time, a good natural exercise environment also helps students to develop external natural atmosphere attachment psychology (R. H. Liu et al., 2017). According to environmental psychology, people’s emotional connection to a specific place causes them to develop a sense of place, and that feeling forms a universal, broad, and deep attachment relationship between people and places (i.e., place attachment) and affects the development of individual socialization (Zhou et al., 2010). In short, people’s spatial perception of the natural environment enriches the individual cognitive system and influences behavioral practice by forming a reference basis for behavioral decision-making (Cai et al., 2012). It can be seen that a good natural exercise atmosphere is an important external resource to improve college students’ physical exercise. 

In addition, research reports that the interpersonal atmosphere can provide college students with role models, motivation, incentives, and education for independent exercise (Cheng & Dong, 2018), which is helpful for college students to develop good physical exercise habits (Pan et al., 2010). Especially during the interpersonal sensitivity period, the interpersonal atmosphere can stimulate college students’ exercise intention and help them establish a sense of group identity and behavioral identity in exercise activities, which is an indispensable part of the individual’s social practice. It can also help college students establish self-confidence and self-identity and improve the level of self-determination motivation on the basis of driving self-determination (Zhu & Qiu, 2006). It can be seen that the interpersonal atmosphere is also an important external motivation to stimulate college students’ desire and interest in physical exercise.

### 4.2. Analysis of the Mediating Role of Enjoyment

Regression analyses found enjoyment to be a mediating variable between exercise atmosphere and college students’ physical exercise, which is in line with previous research. For example, the relationship between college students’ exercise atmosphere and leisure physical exercise is mediated by subjective exercise experience (Cheng & Dong, 2018). This claim is consistent with a recent movement in the behavioral sciences to consider affective mechanisms as necessary for effective and lasting behavior change (Dukes et al., 2021). However, the study did not take into account the importance of specific discrete emotions, such as enjoyment. With the rise of emotionalism in behavioral science, enjoyment theory has received exponential attention in the field of individual behavior (Cabanac & Bonniot-Cabanac, 2007). According to this theory, humans have evolved to not only survive by satisfying their most basic needs (e.g., eating, sleeping, and reproducing) but also to maximize pleasure by repeating positive affective experiences in their environments, inspired by this theory and based on strong empirical evidence (Rhodes & Kates, 2015). The most recent model of physical activity proposes that enjoyment plays a key role in shaping decision-making processes related to physical activity (Rhodes et al., 2019).

This mediating role may stem from emotional effects theory, which suggests that people’s assessment of the current situation and recollection of past experiences trigger emotional experiences, which in turn affect behavioral practices (X. W. Li & Wang, 2018). Typically, college students who are able to be satisfied with the atmosphere of the existing exercise environment and who are able to obtain exercise friendship, help, and support from their peers tend to feel a positive and optimistic enjoyment experience during physical exercise (X. E. Wang & Du, 2010) and are able to rationally analyze the promotional efficacy of physical exercise on improving the self, shaping self-concept, and enhancing social adaptation; thus, they are more likely to form positive exercise behavior, engage in diverse forms of exercise, and explore a wide range of exercise content (Cheng & Dong, 2018).

### 4.3. Analysis of the Mediating Role of Exercise Self-Efficacy

Regression analysis found that exercise self-efficacy was a mediating variable between exercise atmosphere and college student physical exercise, which is consistent with the study of Y. Q. Dong et al. (2022). This study concluded that positive family, school, peer, and community factors will improve the exercise atmosphere around the individual, which in turn will increase the level of individual exercise self-efficacy, and the deeper the individual’s perception of exercise will be, which is conducive to the formation of habitual exercise behavior (Y. Q. Dong et al., 2022). This mediating effect further supports social cognitive theory, which suggests that the environment can influence individual behavior through cognition (Bandura, 1977). Exercise atmosphere is a comprehensive environment formed by multiple subjects such as community, family, school, and peers, including the natural environment of physical exercise provided by the community and school, and the interpersonal support environment of parents, teachers, and peers for individual exercise (Cheng & Dong, 2018). Exercise self-efficacy, as a subjective perception of individuals’ abilities (S. Wang et al., 2016), is an important bridge connecting exercise atmosphere and physical exercise. 

The results show that exercise self-efficacy plays a positive role in college students’ physical exercise, indicating that exercise self-efficacy, as an important cognitive resource, has a positive effect on physical exercise promotion, can stimulate individuals’ motivation to engage in physical exercise (F. Wang et al., 2022), helps individuals’ will be based on their own ability to choose suitable exercise tasks and behaviors for themselves, helping individuals to cope with difficulties or setbacks with a calm mindset and stable self-confidence (B. L. Dong et al., 2018). This is an endogenous motivation to guarantee the stability and automation of exercise behaviors (Cheng & Dong, 2018). As stated in social cognitive theory, self-efficacy has a significant impact on behavior, which shapes an individual’s behavioral patterns by influencing his or her motivation, behavioral choices, effort, and persistence (Bandura, 1997). 

### 4.4. Analysis of the Chain Mediating Role of Enjoyment and Exercise Self-Efficacy

The results suggest that enjoyment has an impact on physical exercise through exercise self-efficacy. This finding is consistent with previous research (H. Chen et al., 2017), which demonstrated that the relationship between enjoyment and self-efficacy is well documented (Barnett, 2013). The findings of the current study extend previous research by demonstrating that enjoyment precedes self-efficacy. This finding is consistent with social cognitive theory, which suggests that individuals’ emotional experiences may influence their self-efficacy (Bandura, 1997). When individuals feel enjoyment during exercise, they are more likely to rate their performance positively. This positive feedback mechanism acts directly on an individual’s self-confidence, leading them to believe that they are competent and enjoy exercise activities (Lewis et al., 2016). For example, the positive emotional experience of feeling satisfied after completing a high-intensity exercise prompts individuals to believe that they are capable of continuing and possibly increasing the intensity or difficulty of their exercise in the future (Teixeira et al., 2022). 

Furthermore, our findings are consistent with the results of a longitudinal study that demonstrated that the intervention had a positive impact on high school girls’ enjoyment of physical activity, thereby increasing their self-efficacy. Increased self-efficacy contributes to increased physical activity (Dishman et al., 2005). Finally, two mediating variables have a significant sequential mediating effect between exercise atmosphere and physical exercise. This finding may be of significant value, suggesting that exercise atmosphere can increase college students’ enjoyment of physical exercise, thereby increasing their exercise self-efficacy. Increased exercise self-efficacy increases physical exercise among college students. This finding suggests that enjoyment and exercise self-efficacy are key situational elements that chain-mediated effects between exercise atmosphere and physical exercise. It reveals how environment, emotion, and cognition interact with individual behavior, confirms that positive affective experiences play a key role in shaping decision-making processes related to physical activity (Rhodes & Kates, 2015), and facilitates the integrated application of social-cognitive theory and affective-efficacy theory to exercise promotion.

### 4.5. Analysis of the Moderating Effect of Social Physique Anxiety

The findings suggest that social physique anxiety negatively moderates the effects of exercise atmosphere on enjoyment and exercise self-efficacy, as well as their mediating effects between exercise atmosphere and physical exercise, which reveals how individual self-presentation interacts with the environment and emotion/cognition to influence behavior (Rhodes & Kates, 2015). It also validates the mechanisms by which social physique anxiety affects college students’ physical exercise, expanding previous research on the correlation between social physique anxiety and exercise behavior (Crawford & Eklund, 1994; Xu, 2003; Rothberger, 2014). Individuals with high social physique anxiety are more likely to be distracted during exercise and may be overly concerned with their posture, whether their movements are standardized, and whether others are observing them (Xu, 2003; Frederick & Morrison, 1996), thus reducing enjoyment of the exercise itself. Meanwhile, social physique anxiety diminishes an individual’s exercise self-efficacy by reducing their sense of self-worth and self-confidence (Rothberger et al., 2015), which in turn diminishes their exercise self-efficacy. When individuals are concerned that their fitness is rated poorly by others, they may doubt their ability to exercise and believe that they will not be able to achieve their desired exercise results or goals (Sabiston et al., 2014). This self-doubt and denial can weaken their exercise self-efficacy, making them more likely to abandon their exercise program or reduce their exercise effort (Yan et al., 2019). In addition, individuals with high social physique anxiety tend to worry about others’ negative evaluations of their physiques and intentionally avoid exercise venues and community facilities, as well as interactions with peers around them, which greatly diminishes the positive effects of natural and interpersonal exercise atmospheres on college students’ enjoyment and exercise self-efficacy (Cheng & Dong, 2018; Zhang et al., 2022). This reduced enjoyment and exercise self-efficacy caused by social physique anxiety, in turn, weakens exercise motivation and hinders the persistence and enthusiasm of exercise behavior (Lewis et al., 2016).

The results of the study suggest that social physique anxiety negatively moderates the chain-mediated effects of enjoyment and self-efficacy between exercise atmosphere and physical exercise and that the results of this study are primarily the result of social physique anxiety negatively moderating the effects of exercise atmosphere on enjoyment, further elucidating how individual self-presentation interacts with the environment, emotion, and cognition to influence behavior (Rhodes & Kates, 2015). The dual-process model suggests that two information processing systems of human association and deliberation can process information about external environmental stimuli in a sequential manner. According to the dual-process model (Chu et al., 2020), under high social physique anxiety, college students first process exercise atmosphere coping strategies through the associative system, which activates their exercise memories of previous negative information related to their own physical evaluations, such as stares, evaluations, or comparisons from others. This negative memory interferes with their positive perception of the exercise atmosphere through deliberation processing (Timme et al., 2023), diminishes the pleasure they experience from the exercise environment, and creates doubts and uneasiness about their own ability to exercise, which in turn decreases self-efficacy (Guo et al., 2023), leading to a lack of motivation and confidence when individuals are faced with exercise choices (Rothberger et al., 2015), thus reducing the frequency and intensity of physical exercise. This leads to the conclusion that social physique anxiety is a key self-presenting element that negatively moderates the chain-mediated effects of enjoyment and self-efficacy between exercise atmosphere and physical exercise.

## 5. Implications

First of all, build a culture of exercise life, create a campus exercise atmosphere, and give full play to the aggregation effect of multiple systems such as community, family, school, and peers. Form a material environment, family social support environment, and interpersonal atmosphere conducive to college students’ exercise participation, and enhance college students’ exercise behavior. Secondly, carry out emerging sports programs that are diversified, interesting, and personalized. At the same time, we should also pay attention to the subjective enjoyment experience of college students and understand their exercise needs and feelings through questionnaires and other means, so as to better satisfy their exercise needs. Once again, actively develop sports clubs, encourage social sports organizations to enter the campus, improve students’ interpersonal relationships in exercise and encourage social interactions among exercisers, such as organizing group exercise and sharing exercise tips. This not only enhances the fun of exercise but also promotes mutual motivation and support among exercisers and improves students’ exercise self-efficacy. Finally, take the initiative to cope with and prevent the negative effects of social physique anxiety on college students’ physical exercise. Schools, in conjunction with the media and families, can collaborate to promote the theoretical education of exercise for weight control and body shape maintenance, disseminate scientific knowledge of physical health to college students, and change the widespread, deep-rooted, and preconceived mentality of worrying about others’ negative evaluation of physique; revise and improve the content of mental health education courses to help college students establish a correct perception of body image, and strengthen the cultivation of college students’ ability to regulate their emotions, especially to build a good and inclusive physique. In particular, it is necessary to shape a favorable and inclusive body image atmosphere, focusing on the psychological catharsis and channeling of the psychological pressure and sense of misfortune of college students with social physique anxiety. Encourage college students to accept information about the diversity of body shapes and sizes, and guide college students to transform social physique anxiety into exercise motivation and shaping challenges, as well as provide professional exercise guidance to help college students formulate a scientific and reasonable exercise program, and give them timely feedback and adjustment suggestions in the exercise process. This helps to enhance college students’ enjoyment and self-efficacy and reduce anxiety due to uncertainty and frustration.

## 6. Limitations and Prospects

This study has the following shortcomings: (1) The questionnaire survey method was used in this study, which could not determine the causal paths and long-term effects of the variables; future studies can use longitudinal surveys or rigorous experimental designs to explore the influence paths between the variables. (2) Interpersonal support may be an important factor in the exercise atmosphere, which mainly includes the support of parents, teachers, and peers, etc. The influence of different interpersonal support on college students’ physical exercise may be different. Future studies may compare the effects of different interpersonal support on physical exercise. (3) In terms of mediating mechanisms, this study only explored the mediating effects of enjoyment and exercise self-efficacy, however, these two variables play a partially mediating role between exercise atmosphere and physical exercise, so the mediating effects between the two can be further explored in terms of other affective and cognitive factors. (4) In terms of sample selection, this study still has limitations in terms of geographic selection and categorization of college students, such as not including college students from different provinces, ages, physical activity levels, educational paths, and collegiate student-athletes, and it needs to be further refined in these areas in the future.

## 7. Conclusions

Exercise atmosphere is an external resource for college students to practice physical exercise, which can directly influence college students’ physical exercise and indirectly influence college students’ physical exercise by acting on individual enjoyment and exercise self-efficacy. This suggests that colleges need to design exercise promotion strategies from the dual paths of environmental optimization (e.g., improved facilities, peer motivation) and psychological empowerment (e.g., improved enjoyment and confidence) to avoid the limitations of a single means. In addition, social physical anxiety negatively moderated the effects of exercise atmosphere on enjoyment and exercise self-efficacy, and negatively moderated the mediating effects of enjoyment and exercise self-efficacy between exercise climate and college students’ physical exercise. This finding reveals that the effects of exercise atmosphere on exercise behavior are not universal but are moderated by individual psychological traits (e.g., social body anxiety). Future interventions need to balance environmental optimization and psychological mitigation, especially focusing on the special needs of high-anxiety groups, in order to more effectively enhance the overall exercise participation of college students.

## Figures and Tables

**Table 1:** Statistics of Pearson Correlation Coefficient.

	M ± SD	1	2	3	4	5	6	7	8	9	10	11
1.Gender	0.60 ± 0.49	1										
2.Grade	0.38 ± 0.49	0.106 **	1									
3.RR	0.47 ± 0.50	−0.028	−0.041	1								
4.PF	3.14 ± 1.04	0.159 **	0.019	−0.087 **	1							
5.BMI	20.84 ± 3.65	−0.162 **	−0.023	0.022	−0.206 **	1						
6.SES	−0.05 ± 2.98	−0.108 **	−0.068 *	0.413 **	−0.067 *	0.033	1					
7.SPA	41.49 ± 9.00	0.167 **	0.002	−0.039	−0.009	0.073 **	−0.105 **	1				
8.EN	12.52 ± 3.55	−0.175 **	−0.013	−0.020	0.112 **	0.052	0.007	−0.109 **	1			
9.ES	18.66 ± 5.40	−0.235 **	−0.009	−0.035	0.123 **	0.015	0.058 *	−0.168 **	0.553 **	1		
10.EA	37.87 ± 6.91	−0.021	0.045	0.021	0.092 **	0.020	0.037	−0.103 **	0.510 **	0.390 **	1	
11.PS	18.40 ± 17.00	−0.270 **	−0.015	0.019	0.132 **	0.049	0.081 **	−0.140 **	0.345 **	0.348 **	0.222 **	1

Note: * *p* < 0.05, ** *p* < 0.01; RR, Registered residence; PF, Physical fitness test; SPA, social physique anxiety; EN, enjoyment; ES, exercise self-efficacy; EA, exercise atmosphere; PS, physical exercise. (the same below).

**Table 2:** Regression analysis of the multiple mediation model.

	EN			ES			PS		
	β	SE	t	β	SE	t	β	SE	t
Gender	−0.188	0.025	−7.420 ***	−0.165	0.025	−6.554 ***	−0.206	0.027	−7.519 ***
PF	0.112	0.026	4.405 ***	0.081	0.025	3.258 **	0.129	0.027	4.803 ***
BMI	0.034	0.029	1.160	−0.020	0.029	−0.694	0.035	0.031	1.144
SES	−0.022	0.025	−0.885	0.038	0.024	1.599	0.054	0.026	2.091 *
EA	0.494	0.025	20.129 ***	0.155	0.028	5.591 ***	0.064	0.030	2.129 *
EN				0.441	0.029	15.480 ***	0.155	0.034	4.636 ***
ES							0.146	0.031	4.665 ***
R2	0.305			0.356			0.203		
F	102.081 ***			106.997 ***			42.173 ***		

Note: * *p* < 0.05, ** *p* < 0.01, *** *p* < 0.001.

**Table 3:** Analysis of the mediating effects of EN and ES.

	Effect Size	BootSE	Boot95%CI	
			Lower	Upper
Total effect	0.195	0.028	0.139	0.249
Direct effect	0.064	0.029	0.008	0.123
Total mediating effect	0.131	0.020	0.094	0.171
Mediating effect1	0.076	0.020	0.039	0.115
Mediating effect2	0.023	0.007	0.010	0.039
Mediating effect3	0.032	0.008	0.018	0.048

**Table 4:** Regression analysis of moderated multiple mediation models.

	EN			ES			PS		
	β	SE	t	β	SE	t	β	SE	t
Gender	−0.181	0.026	−7.050 ***	−0.150	0.025	−5.956 ***	-0.206	0.027	−7.519 ***
PF	0.113	0.026	4.434 ***	0.083	0.025	3.359 **	0.129	0.027	4.803 ***
BMI	0.035	0.030	1.173	−0.014	0.029	−0.492	0.035	0.031	1.144
SES	−0.024	0.025	−0.957	0.032	0.024	1.358	0.054	0.026	2.091 *
EA	0.484	0.025	19.503 ***	0.142	0.028	5.130 ***	0.064	0.030	2.129 *
EN				0.431	0.028	15.220 ***	0.155	0.034	4.636 ***
ES							0.146	0.031	4.665 ***
SPA	−0.022	0.025	−0.889	−0.070	0.024	−2.897 **			
EA × SPA	−0.051	0.022	−2.342 *	−0.069	0.021	−3.312 **			
*R* ^2^	0.310			0.369			0.203		
*F*	74.231 ***			84.472 ***			42.173 ***		

Note: * *p* < 0.05, ** *p* < 0.01, *** *p* < 0.001.

**Table 5:** Moderated mediation effects (Bootstrap = 5000).

Moderated Path	SPA	Effect Size	BootSE	Boot95%CI	
				Lower	Upper
Path1: EA-EN-PS	High (M + 1SD)	0.067	0.018	0.034	0.104
	Low (M − 1SD)	0.083	0.022	0.042	0.127
	Between-group difference	−0.016	0.009	−0.035	−0.001
Path2: EA-ES-PS	High (M + 1SD)	0.011	0.007	−0.002	0.025
	Low (M − 1SD)	0.031	0.010	0.014	0.052
	Between-group difference	−0.020	0.009	−0.041	−0.005
Path3: EA-EN-ES-PS	High (M + 1SD)	0.027	0.007	0.014	0.043
	Low (M − 1SD)	0.034	0.009	0.018	0.052
	Between-group difference	−0.007	0.004	−0.015	−0.001

## Data Availability

The datasets used and/or analyzed during the current study are available from the corresponding author on reasonable request.
